# p63 is required beside p53 for PERP-mediated apoptosis in uveal melanoma

**DOI:** 10.1038/bjc.2016.269

**Published:** 2016-09-01

**Authors:** Raheela Awais, David G Spiller, Michael R H White, Luminita Paraoan

**Affiliations:** 1 Department of Eye and Vision Science, Institute of Ageing and Chronic Disease, University of Liverpool, William Henry Duncan Building, 6 West Derby Street, Liverpool L7 8TX, UK; 2 Systems Microscopy Centre, Faculty of Life Sciences, University of Manchester, Manchester M13 9PT, UK

**Keywords:** p63, apoptosis, PERP, p53, uveal melanoma, chromosome 3, monosomy 3

## Abstract

**Background:**

PERP (p53 apoptosis effector related to PMP-22), a transcriptional target of p53, is downregulated and contributes to the impairment of apoptosis in uveal melanoma (UM). Intriguingly, PERP is not induced in UM despite functional p53. p63, located on chromosome 3, which is characteristically altered in high-risk UM, can transactivate PERP. Here, we determine the functional role of p63 expression in the initiation of p53/PERP-mediated apoptosis in UM.

**Methods:**

PERP expression was monitored by quantitative PCR (qPCR) and immunoblotting in UM cell lines treated with DNA-damaging agents. The functional role of p63 was assessed by transient expression of p63-turbo GFP (p63-tGFP) in the apoptosis- resistant, 3q-deficient OCM-1 cells. Expression and localisation of p63, PERP and p53, and induction of apoptosis were characterised by qPCR, immunoblotting and live cell confocal microscopy.

**Results:**

PERP expression was significantly downregulated in all UM cell lines. DNA-damaging treatments failed to induce apoptosis and activate PERP in OCM-1 cells, which displayed non-functional levels of p63. Expression of p63-tGFP induced apoptosis with marked increase in PERP expression and associated p53 accumulation.

**Conclusions:**

Lack of p63 contributes to reduced PERP levels and impaired p53-mediated apoptosis in UM. p63 expression is required for PERP-mediated apoptosis in UM.

Uveal melanoma (UM) tumours of high risk have a propensity to metastasise to the liver (Albert, 1997; Kujala *et al*, 2003; Kivela *et al*, 2006) and are characterised by chromosomal abnormalities, including loss of chromosome 3 (monosomy 3) or partial gains of chromosome 1, 6 or 8 (Aalto *et al*, 2001). Despite various advancements in the diagnosis and treatment of UM over the past few decades, disease-related mortality is still high, with median overall mortality rate of 6 months postdiagnosis of metastasis (Triozzi *et al*, 2008; Spagnolo *et al*, 2012). Progress in treatment is hindered by the marked resistance of the aggressive type of UM to conventional chemotherapy and radiotherapy. These therapeutic agents act primarily by activating the cell death pathways. Failure to respond to these agents implies that the resistance to apoptosis is a prominent feature of UM. Therefore, the identification of specific molecular mechanisms underlying the evasion of apoptosis is critical for providing the basis for targeted approaches to eventually overcome the apoptosis resistance in UM.

Among the huge diversity of genes implicated in tumour development, the transcription factor p53, together with its complex signalling cascade, stands out as a master regulator of cell survival/death pathways. Approximately 50% of human solid tumours present direct p53 mutations and the majority of adult cancers present impairment of the p53 signalling pathway and/or its downstream targets (Levine, 1997; Vogelstein *et al*, 2000). The latter scenario appears to be the mechanism for evasion of apoptosis that is used by UM since there is no consistent evidence of frequent direct p53 inactivation, mutation or altered signalling upstream to p53 in these tumours (Brantley and Harbour, 2000; Sun *et al*, 2005). The overall signalling impairment through p53 pathway (Mooy *et al*, 1995; Chana *et al*, 1999; Imesch and Albert, 1997; Brantley and Harbour, 2000) points therefore towards functional defects downstream of p53. We have previously identified the p53 apoptosis effector related to PMP-22 (PERP) as one such defect that has an important role in the regulation of apoptosis in UM cells (Paraoan *et al*, 2006). Transcriptional downregulation of the PERP gene was also reported in metastasising cutaneous melanoma, pancreas and mammary carcinoma cell lines, as well as in tumours of the ovary cervix, uterus and breast (Hildebrandt *et al*, 2000)

PERP was initially identified as a p53 transcriptional target that is distinctively induced during apoptosis in response to DNA damage or UVC treatment but not during cell cycle arrest (Attardi *et al*, 2000). The expression of PERP is significantly downregulated at the transcriptional and protein level in the aggressive, highly metastatic monosomy 3-type UM tumours compared with disomy 3-type UM tumours (Paraoan *et al*, 2006). We previously showed that induced expression of PERP is sufficient to initiate p53-dependent apoptosis in UM cells *in vitro*, which involves the upregulation of death receptor 4 or TNF-related apoptosis-inducing ligand (TRAIL) receptor 1 and activation of caspase-8 (Davies *et al*, 2009). We also demonstrated that elevated PERP expression significantly enhances the active levels of its own transcriptional regulator, p53, which subsequently promotes apoptosis by undergoing apoptosis-specific phosphorylation and by transcriptionally activating specific proapoptotic genes (Davies *et al*, 2011). Taken together, these studies indicate that the endogenous levels of PERP can influence both upstream and downstream regulation of the p53 pathway and place PERP at a key signalling junction of p53-mediated apoptosis in UM, thus rendering it a potential target for developing apoptosis-based cancer therapies.

PERP contains several p53 binding sites in its promoter region (Attardi *et al*, 2000; Reczek *et al*, 2003). However, the normal level of p53 fails to induce PERP transcription in UM cells, suggesting that there may be additional regulatory elements required to initiate PERP expression either by cooperating with p53 or directly interacting with the PERP promoter. Previous studies showed that p53 requires its homologues p63 and p73 to induce apoptosis in response to DNA damage (Flores *et al*, 2002). Indeed, the induction of many target genes is shared among these three family members (Jost *et al*, 1997; Yang *et al*, 1998), which are interdependent in eliciting the apoptotic response and suppression of tumourigenesis (Flores *et al*, 2002, 2005). Hence, PERP may also be transactivated by p63 through the p53/p63 consensus element in intron 1, the major p53-reponsive site identified in PERP (Ihrie *et al*, 2005).

p63, located at chromosome 3q27-29, is primarily recognised as a global regulator of epithelial development, integrity and homeostasis. p63 knockout mice manifest marked absence of limbs, skin, hair, mammary, lacrymal and salivary glands owing to failure of these epithelia to develop during gestation (Mills *et al*, 1999; Yang *et al*, 1999). PERP expression governed by p63 was mainly associated with the stratified epithelium development and maintaining the epithelial integrity by promoting desmosomal cell–cell adhesion processes in which PERP requires direct activation by p63 (Ihrie *et al*, 2005; Ihrie and Attardi, 2005). The functional association of PERP and p63 has been extensively studied in the context of epithelial integrity, but there is scarce information on the possible interconnection of p63 and PERP in apoptosis. The only such study to our knowledge is by Flores *et al* (2002), in which p63 was shown to bind to PERP promoter after DNA damage in p53^−/−^ E1A mouse embryonic fibroblasts, suggesting that p63 and p73 might regulate the ability of p53 to bind at certain selected promoters following DNA damage.

The present study investigated for the first time the role of p63 in the PERP-mediated apoptotic pathway in UM. Our findings indicate that lack of functional p63 (likely due to loss of chromosome 3 in monosomy 3-type UM tumours) is a major cause of significantly reduced PERP levels and of impairment of p53-mediated apoptotic pathway in UM. In addition, our findings establish that the introduction of p63 by exogenous expression leads to significantly increased susceptibility of UM cells to apoptosis. We present evidence that p63 expression in UM cells results in transcriptional and translational activation of endogenous PERP, which in turn drives apoptosis in UM. Finally, we show that apoptosis caused by exogenous p63 expression requires the activation of p53. The results provide novel insights into the mechanisms underlying the apoptotic resistance in tumours with reduced PERP expression.

## Materials and methods

### Cell culture

The human UM cell lines used in this study, MEL 290, MEL285, MEL202, 92-1 and OCM-1, kindly provided by Dr Martine Jager (University Hospital Leiden, Leiden, The Netherlands) (Maat *et al*, 2008) and Dr Dan Albert (University of Wisconsin-Madison, Madison, WI, USA) (van Ginkel *et al*, 2008), were cultured in RPMI-1640 with 2 mM L-glutamine and 25 mM HEPES (Gibco Life Technologies, Paisley, UK) supplemented with 10% (v v^−1^) heat-inactivated foetal calf serum (Biosera, East Sussex, UK), 1 mM sodium pyruvate and 1% (v v^−1^) non-essential amino acids (Sigma-Aldrich, Dorset, UK). ARPE-19 cells (ATCC, CRL-2302, LGC Standards, Middlesex, UK) were cultured in DMEM-F12 supplemented with L-glutamine (Sigma-Aldrich).

### TRAIL and UV treatment of OCM-1 cells

Treatment with TRAIL was carried out on subconfluent cultures (50 000 cells per well in 12-well plates) with fresh medium at the following concentrations: 300, 450 and 600 ng ml^−1^. The viability of cells treated with TRAIL was assessed using phase-contrast microscopy. OCM-1 cells were subjected to UV irradiation at the dose of 15 mJ cm^−2^ using CL-1000 UV crosslinker UVP. The cells were collected and lysed at 3, 24 and 48 h post-treatment for immunoblotting analysis.

### Cell transfection

Transient transfections were carried out using a p63-turbo GFP (p63-tGFP) expression plasmid (Origene, Rockville, MD, USA), which encodes full-length human protein p63 (NM_003722) transcript variant 1 tagged with tGFP. OCM-1 cells were seeded at a density of 2 × 10^5^ cells per well in 6-well plates for protein and RNA extraction and in 35-mm glass-bottomed (Iwaki, Appleton woods, Sellyoak, Birmingham, UK) dishes for confocal imaging. Transfections were performed with Turbofect *in vitro* transfection reagent (Thermo Scientific, Paisley, UK) with 3 *μ*g of DNA and 6.0 *μ*l Turbofect per well or per dish according to the manufacturer's instructions. Cells were lysed and collected for RNA extraction and western blotting at 16, 24, 48 and 72 h post-transfection. Time-lapse cell imaging was performed 24 h post-transfection.

### Real-time quantitative PCR

RNA was purified using RNeasy Kit (Qiagen Ltd, Manchester, UK). cDNA was synthesised using 1 *μ*g of RNA and SuperScript VILO cDNA Synthesis Kit (Invitrogen, Paisley, UK). Real-time quantitative PCR (qPCR) was performed with gene-specific primer sets for PERP, p63 and GAPDH (Table 1) using a Stratagene MX3000P qPCR System (Stratagene, La Jolla, CA, USA) and MESA Blue qPCR Kit for SYBR Green assay (Eurogentec, Southampton, UK) according to the manufacturer's instructions. Genome-wide validated primer sequences (Origene) were used to avoid any cross-amplification between p53, p63 and p73 (which have a high degree of homology).

### Laser scanning confocal fluorescence microscopy of live transfected cells

OCM-1 cells cultured in Iwaki dishes were transfected with p63-tGFP using Turbofect transfection reagent as described above. Annexin-V and propidium iodide (PI) were added sequentially 24 h post-transfection and live cell imaging was performed on cells maintained in a humidified incubator (37 °C, 5% CO_2_) using a Zeiss LSM 510 microscope with a Fluar × 20/0.75 UV objective (Carl Zeiss, Jena, Germany). Excitation of GFP was carried out with 488-nm argon laser and emitted light was collected through 505–550 nm bandpass filters. Propidium iodide was excited at 543 nm with a HeNe laser and detected at 565–615 nm, whereas the Alexa Flour 647 Annexin-V-conjugated dye was excited at 633 nm and detected at 651–694 nm. Data acquisition and analysis were carried out with Zeiss LSM 510 software, AIM version 3.2 (Carl Zeiss).

### Determination of cell viability and apoptosis detection assay

Apoptosis was detected by incubating the cells with Alexa Fluor 647 Annexin-V-conjugated dye (1:500 dilution) as an indicator of early-stage apoptosis and PI (2 *μ*g ml^−1^) to identify cells with lost plasma membrane integrity. Cell counts were obtained from 30 separate fields of view at 24–48 h after transfection. The percentage of dead cells was determined for transfected or non-transfected cells in all selected fields.

### Immunoblotting

Cultured OCM-1 cells were harvested at 16, 24, 48 and 72 h after transfection with p63-tGFP and resuspended in lysis buffer (0.128 M *β*-mercaptoethanol, 40 mM Tris, 10% glycerol, 1% SDS and 0.01% bromophenol blue; all materials from Sigma-Aldrich) supplemented with protease and phosphatase inhibitors (PhoStop and cOmplete PIC; Roche, West Sussex, UK). The proteins were separated by 12% gel electrophoresis (10% for the detection of p63-tGFP because of its larger size, 103 kDa), transferred to nitrocellulose membrane and probed with respective primary antibodies: PERP (ab5986; Abcam, Cambridge, UK), p53 (P-6874; Sigma-Aldrich) and GAPDH (ab8245; Abcam). Sequential probing of the blots was carried out after incubation in stripping solution at 65 °C for 30 min, followed by 2 × 10 min washes in TBS-T before blocking in 5% milk/TBS-T and reprobing. Immunocomplexes were detected with horseradish peroxidase-conjugated secondary antibody (Sigma-Aldrich) and chemiluminescence (Amersham ECL, Little Chalfont, UK). Two types of p63 antibodies were used: anti-p63 (ab53039; Abcam) detecting all p63 proteins and anti-p63 (ab97865; Abcam) detecting the p63 transcript variant 1 (77 kDa).

## Results

### PERP is downregulated in UM cell lines

**Figure 1:**
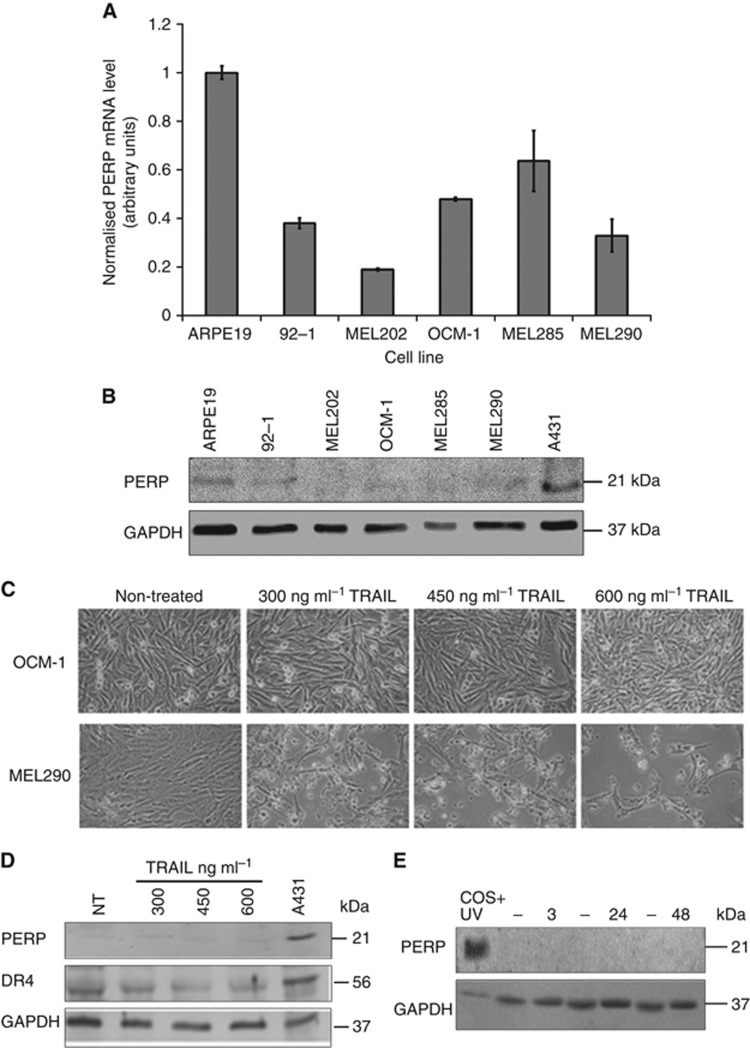
Endogenous PERP expression in UM cells and differential response following TRAIL and UV treatment. Low PERP expression at the (**A**) transcriptional and (**B**) protein level in all UM cell lines tested indicated downregulation of PERP in these cells. (**A**) endogenous PERP mRNA levels determined by qPCR normalised to the level of glyceraldehyde-3-phosphate dehydrogenase (GAPDH) are shown relative to the normalised PERP mRNA level in ARPE19 cells (given an arbitrary value of 1). The mean of three independent experiments along with s.d. is presented. (**B**) Western blot showing relative protein levels of endogenous PERP in UM cell lysates. A431 cell lysate (human epithelial carcinoma whole-cell lysate; Abcam; ab7909) served as a positive control for the PERP antibody. (**C**) Differential response of OCM-1 and MEL290 cells to the TRAIL treatment for 36 h at the concentration of 300, 450 and 600 ng ml^−1^. OCM-1 cells remained viable with normal cell morphology, even at the highest TRAIL concentration. (**D**) Western blots indicating the level of PERP and DR4 proteins in OCM-1 cell lysates following treatment (36 h) with various concentrations of TRAIL. Non-treated (NT) cells served as control for the TRAIL treatment. (**E**) Following UV irradiation at the dose of 15 mJ cm^−2^, OCM-1 cells were collected and lysed at 3, 24 and 48 h post-treatment for immunoblot analysis using PERP antibody. UV-treated Cos-7 cell lysate (Monkey kidney whole-cell lysate; Santa Cruz Biotechnology, Insight Biotechnology Ltd, Middlesex, UK; sc-24666) served as a positive control. GAPDH was used as the loading control in all experiments.

Endogenous PERP mRNA and protein levels were determined in five UM cell lines (92-1, MEL202, OCM-1, MEL 285 and MEL290) and ARPE-19 (as a non-cancer reference cell line). Levels of PERP mRNA were reduced in all UM cell lines compared with ARPE-19 (Figure 1A). Although some variation of PERP mRNA levels was detected between different UM cell lines, this was not statistically significant. The level of PERP protein was also low in all UM cell lines consistent with the transcriptional profile of PERP gene in these cells (Figure 1B).

### PERP is not activated by TRAIL or UV treatment in UM cells

p53 is a transcriptional regulator of PERP. PERP activation is associated with p53-driven upregulation of DR4 and engagement of the caspase-8 apoptotic pathway (Davies *et al*, 2009, 2011). We investigated whether activation of p53 apoptotic pathways by death-inducing ligands, such as TRAIL or UV-induced DNA damage lead to the upregulation of PERP and subsequent apoptosis. First, UM cell viability following treatment with TRAIL (over the range 300–600 ng ml^−1^) was assessed by phase-contrast microscopy. Among all cell lines, MEL290 cells were found to be most sensitive to TRAIL (Figure 1C). OCM-1 cells, on the other hand, were most resistant and showed minimal cell death (<5%), even at a dose of 600 ng ml^−1^. Consequently, we used OCM-1 cells, which also contain marked chromosome 3 aberrations, as a model for the aggressive type, apoptosis-resistant UM cells, which are unresponsive to p53 pathway-targeted chemo- and radiotherapy treatments. To further analyse whether the failure in initiating apoptosis after TRAIL treatment is associated with lack of upregulation of PERP, TRAIL-treated OCM-1 cells were lysed and the level of PERP protein was determined. The treatment with TRAIL at all concentrations tested did not lead to any increase in the levels of either PERP or the functionally associated DR4 (Figure 1D), indicating the inability of these cells to engage apoptotic pathways in response to these death-inducing ligands.

The expression of PERP in OCM-1 cells was also investigated following UV irradiation. Similar to TRAIL treatment, PERP was not induced after UV treatment (Figure 1E), indicating the lack of response of these cells to the UV-triggered p53-dependent cell death.

### p63 is significantly downregulated in UM cell lines

**Figure 2:**
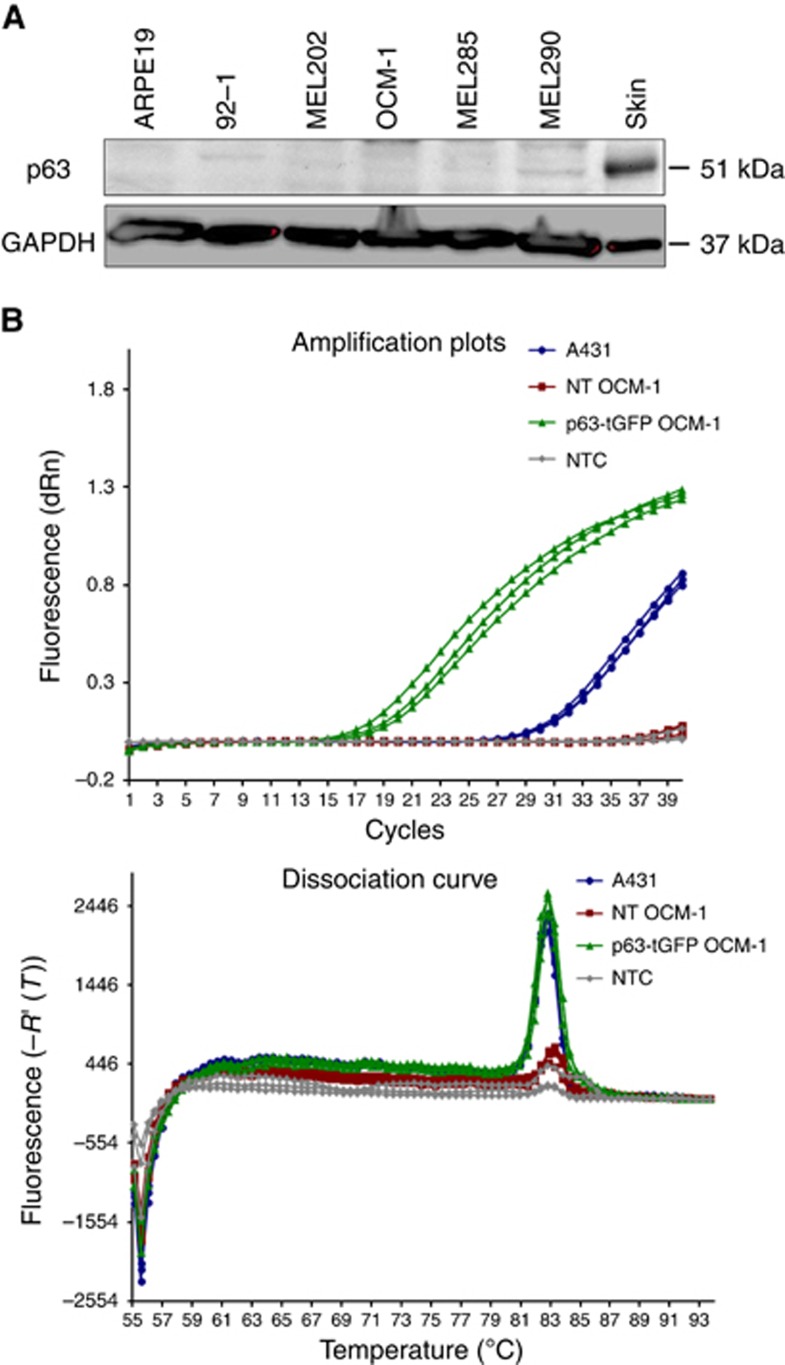
p63 is significantly down-regulated in UM cell lines. (**A**) Representative western blot showing relative protein levels of endogenous p63 protein in UM cell lysates. Skin cell lysate served as a positive control for p63 antibody. (**B**) mRNA was extracted from non-transfected (NT) OCM-1 as well as from A431 cells and p63-tGFP-transfected OCM-1 cells used as positive controls for p63 expression. Quantitative PCR was performed to assess mRNA levels by analysing the amplification plots and dissociation curves for each of the three types of cells. NTC, non-template control.

The expression of endogenous p63 protein was assessed in the same set of UM cell lines showing undetectable levels in all with the exception of MEL290 cells, where a faint band was detected (Figure 2A). Notably, all these cell lines have been reported to have several complex chromosomal aberrations, ranging from partial deletions to the net loss of chromosome 3q in the case of OCM-1 (Luyten *et al*, 1996).

Considering the chromosomal location of p63 as 3q27-28 and the net loss of 3q associated with OCM-1, these cells were further screened for endogenous p63 mRNA expression by qPCR using A431 cells and p63-tGFP-transfected OCM-1 as a positive control for p63 expression. A cT value was detected in non-transfected (NT) OCM-1 cells, indicating the presence of endogenous p63 transcript. This was, however, very high compared with A431 cells, indicating the presence of only trace amounts of p63 mRNA in OCM-1 cells (Figure 2B). The presence of low-level endogenous p63 transcript in NT OCM-1 was further confirmed by the analysis of the dissociation curve in which the single specific peak was identified for NT OCM-1 A431 and p63-transfected OCM-1. However, the peak height, which represents the amount of product was significantly low in NT OCM-1. Both the high cT value and low peak height indicated the presence of very low p63 mRNA levels in OCM-1 cells, negligible in comparison with A431 cells expressing full-length p63. Taken together, these results indicate that p63 is significantly downregulated both at the transcriptional and translational level in UM cell lines.

### p63 expression induces apoptosis in UM (OCM-1) cells

**Figure 3:**
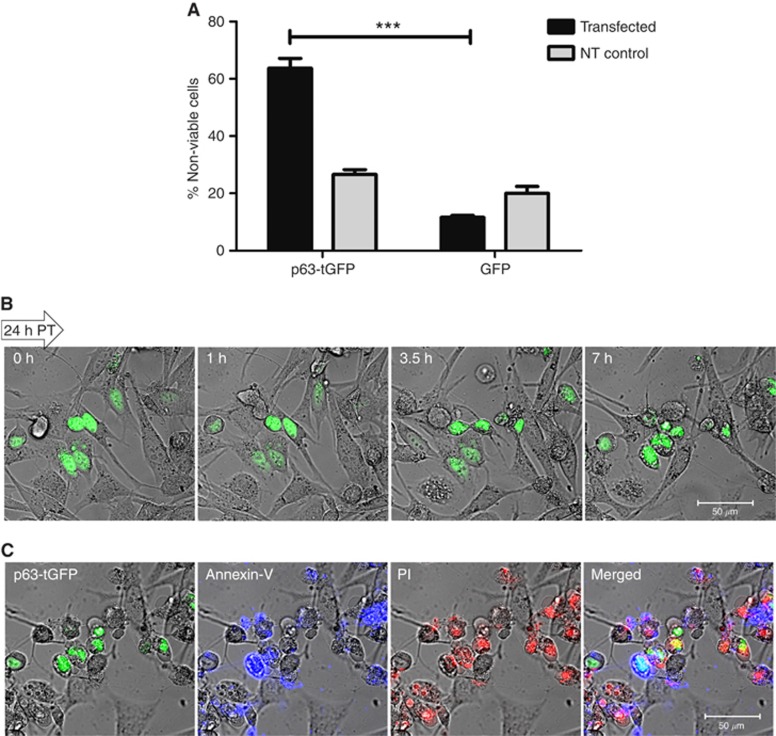
Effect of p63 expression on OCM-1 cell viability. (**A**) OCM-1 cells were transiently transfected with p63-tGFP and the number of viable and nonviable cells in 10 different fields per dish (total number of fields analysed=30) was counted between 24 and 48 h after transfection using Annexin- V and PI as indicators of apoptosis. The mean percentage of transfected (or non-transfected) cells dead in the transfected or NT cell population with standard deviation from three independent transfections is shown (Students's *t*-test, ***P⩽0.0001) compared with GFP-only transfected cells). (**B**) p63-tGFP transiently transfected OCM-1 cells were monitored through time-lapse confocal imaging for 24 h starting 24 h post-transfection, with images captured every 10 min. p63-tGFP-transfected cells showing nuclear localisation of p63 undergoing apoptosis are shown over time alongside non-transfected cells within the same cell population. (**C**) OCM-1 cells expressing p63-tGFP showing positive Annexin-V binding (blue) indicate early apoptosis, whereas the PI staining (red) highlights the cells that have lost membrane integrity.

To initiate the functional studies of p63 in OCM-1 cells, we determined the effect of induced p63 expression on cell viability. For this purpose, cells were transiently transfected with p63-tGFP and the cell viability assessed by Annexin-V binding and PI staining between 24 and 48 h after transfection. Cell death was found to be significantly higher in the cells transfected with p63-tGFP (64.78±8.17%) compared with non-transfected cells (26.6±2.8%), or cells transfected with GFP only (Student's *t*-test, ***P⩽0.0001; Figure 3A). To confirm the p63-dependent apoptosis in real time at the single-cell level, time-lapse imaging was performed on p63-tGFP-transfected OCM-1 cells stained with Annexin-V and PI. The images confirmed the nuclear localisation of p63 and onset of apoptosis in almost all p63-transfected cells in a given field during the observation time (Figure 3B), whereas majority of the non-transfected cells surrounding the transfected apoptotic cells within the same cell population remained viable with normal appearance (non-fluorescent cells; Figure 3B). The p63-transfected apoptotic cells showed positive Annexin-V binding (blue) and PI staining (red), thus confirming the apoptotic characteristics of these cells (Figure 3C).

### p63-led apoptosis involves transcriptional upregulation of endogenous PERP

**Figure 4:**
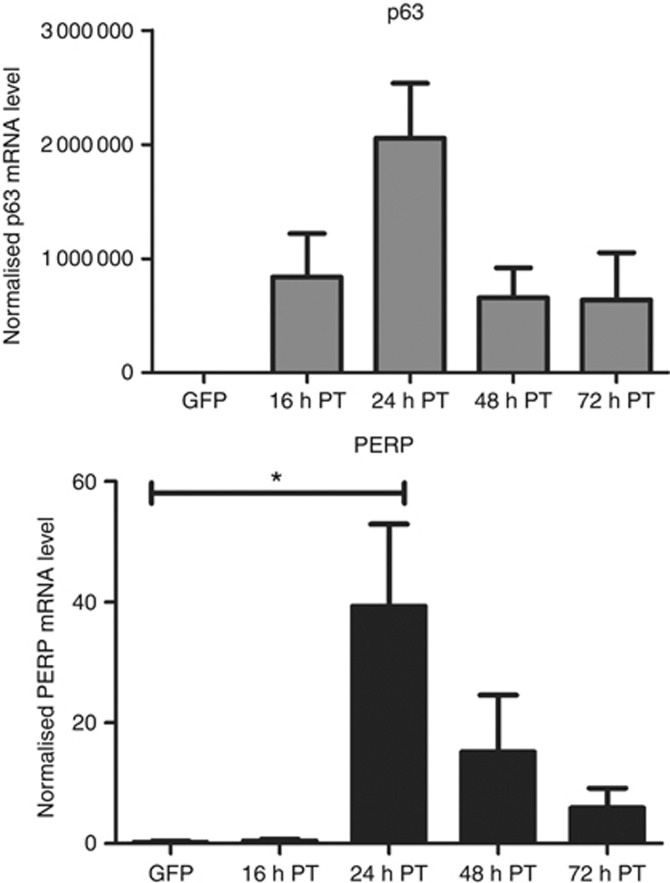
Increased levels of p63 expression lead to the upregulation of PERP mRNA.Total RNA was extracted from OCM-1 cells transiently transfected with GFP-only or p63-tGFP at the indicated times post-transfection (PT). p63 and PERP mRNA levels were determined by qPCR and were normalised to the endogenous level of glyceraldehyde-3-phosphate dehydrogenase (GAPDH). The mean of three independent experiments along with s.d. is presented. Significance was evaluated using Student's *t*-test, *P⩽0.04, compared with GFP-only-transfected cells.

As PERP is an apoptotic effector and a direct transcriptional target of p63, we tested whether PERP could be induced by p63 during the onset of apoptosis in OCM-1 cells. A significant increase in PERP mRNA compared with GFP control (Student's *t*-test, *P⩽ 0.04) was observed at 16–24 h post-transfection when OCM-1 cells expressed p63-tGFP (Figure 4). The pattern of PERP mRNA expression coincided with that observed for p63 mRNA, suggesting that PERP transcription was driven by p63 expression.

### Induction of p63 expression is correlated with activation of p53-mediated PERP apoptosis

**Figure 5:**
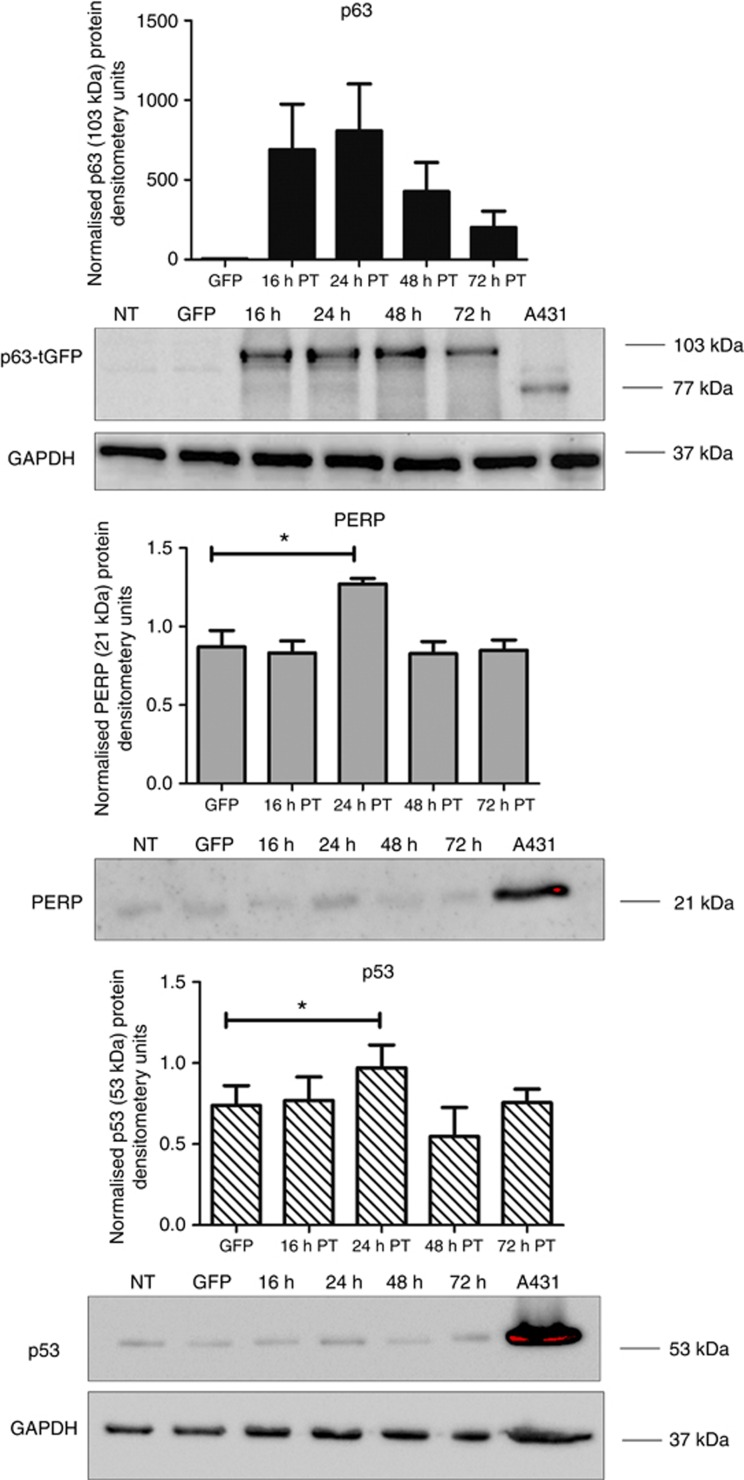
Elevated p63 expression leads to increase in endogenous PERP and p53 protein levels in OCM-1 cells. Cell lysates were prepared at 16, 24, 48 and 72 h post-transfection (PT) with p63-tGFP and analysed by western blotting alongside NT and GFP-only transfected cells that served as controls. A431 cell lysates served as a positive control for p63, p53 and PERP antibodies. p63, PERP and p53 proteins were detected with appropriate antibodies and their relative levels were quantified by densitometry and normalised to glyceraldehyde-3-phosphate dehydrogenase (GAPDH). The mean of three independent experiments along with s.d. is presented. Significance was evaluated with paired *t*-test, compared with GFP-only transfected cells. *P⩽0.03 (PERP) and *P⩽0.02 p53.

**Figure 6:**
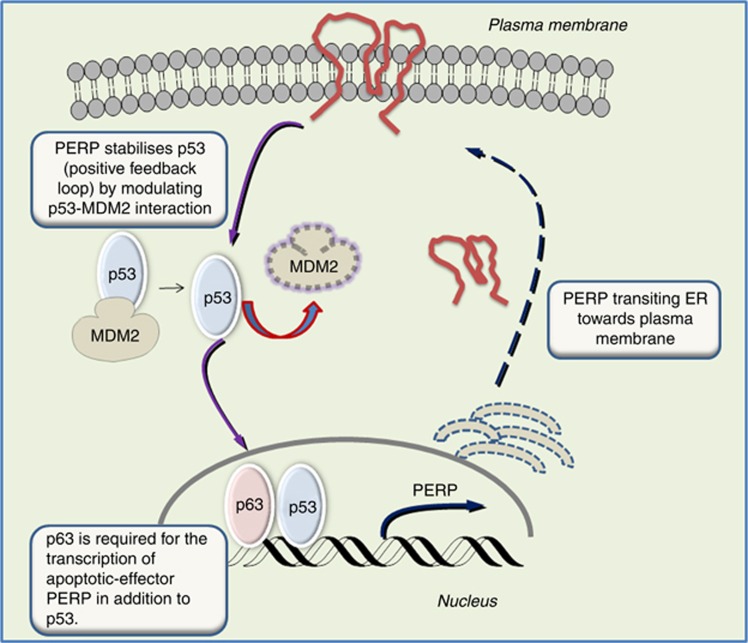
Schematic representation of the involvement of p63 alongside p53 in the transcriptional regulation of PERP with role in initiation of apoptosis in UM. PERP requires direct activation by p63, not only for cell–cell adhesion processes but also for apoptotic functions as well. As previously shown (Davies *et* al, 2011), PERP expression subsequently leads to p53 stabilisation, most likely through a process involving disruption of the interaction between p53 and its negative regulator MDM2.

To determine whether the observed PERP-associated cell death following p63 overexpression involved activation or accumulation of p53, the OCM-1 cells transiently transfected with p63-tGFP were analysed from 16 to 72 h post-transfection (Figure 5). After p63-tGFP expression, a clear increase in the p53 protein level was observed after 24 h compared with GFP-only expressing cells (paired *t*-test, *P⩽0.02). This was also associated with a concurrent increase in PERP levels (*P⩽0.03). The kinetics of PERP and p53 expression were both associated with the p63 expression levels, with the maximum induction of PERP and p53 at the time when p63 expression was at peak levels after 24 h. They then gradually reduced between 48 and 72 h. Taken together, these data indicate that the accumulation of p53 protein is subsequent to induction of p63 expression, which is likely to be mediated through the positive feedback loop by which PERP stabilises p53 (a schematic diagram of the proposed model is presented in Figure 6) (Davies *et al*, 2011).

## Discussion

In UM, the p53 pathway is not affected through p53 mutations or alterations of p53 protein levels, but it is functionally impaired downstream of p53 (Kishore *et al*, 1996; Brantley and Harbour, 2000; Sun *et al*, 2005). Significant downregulation of the p53 effector PERP in primary UM tumours suggests its involvement in a mechanism that allows these tumours to acquire resistance to apoptosis (Paraoan *et al*, 2006). The signalling pathway upstream of p53 and downstream of PERP appears to be intact and functional in UM (Davies *et al*, 2009, 2011), but impairment appears to occur at some stage between p53 and PERP, which is unexpected as p53 is a direct transcriptional regulator of PERP (Attardi *et al*, 2000). These data therefore suggest the involvement of an additional regulator in this process.

p63 is a p53 family member that is a recognised transcriptional regulator of PERP (Ihrie *et al*, 2005) and is known to cooperate functionally with p53 in apoptosis (Yang *et al*, 1998; Flores *et al*, 2002). Importantly, p63 is located on chromosome 3 (Yang *et al*, 1998), where abnormalities are strongly associated with the aggressive type (monosomy 3) of UM. Considering all these factors, we investigated the possible role of p63 as a bridging component between p53 and PERP in the apoptotic pathway in UM cells. The present study demonstrates that p63 has a crucial role in triggering apoptosis in UM cells. This process is also mediated by restoration of transcription and translation of PERP, which is inactive or significantly downregulated in UM primary tumours (Paraoan *et al*, 2006) and in UM cell lines (current study). Our findings suggest that p63 accomplishes its apoptotic role in a p53-dependent manner as supported by the observation of p53 accumulation following exogenous p63 expression.

All UM cell lines used in this study displayed significantly reduced mRNA and protein levels of PERP. This result is in line with our previous finding of downregulation of PERP expression at both the transcriptional and protein level in primary tumour specimens (Paraoan *et al*, 2006). We previously showed that the cell death machinery downstream of PERP that is required for apoptosis in UM cells is responsive to a threshold level of PERP (Davies *et al*, 2009, 2011), which implies that the reduced levels of PERP observed here might not be the result of loss of heterozygosity, but instead might be due to the absence of the regulatory factors needed to activate PERP.

The strong positive association between PERP protein levels and increased levels of cleaved caspase-8 forms, as well as the cleavage of Bid protein (Davies *et al*, 2009), suggested that PERP might transduce its signal through the activation of an extrinsic apoptotic pathway. This p53-dependent pathway involves the engagement of DR4/DR5 by ligands including TRAIL and the formation of the death-inducing signalling complex that leads to the activation of caspases (including caspase-8 and caspase-3), which in turn induce apoptosis (Ashkenazi and Dixit, 1998). Strong induction of PERP has also been reported in response to UVC treatment (Attardi *et al*, 2000). In our study, specific death-inducing stimuli for this pathway did not induce apoptosis or alter the PERP levels in OCM-1 cells. These findings implicate that physiological activation of the canonical p53 apoptotic pathway alone was not sufficient to activate PERP or induce apoptosis.

The gene encoding p63 has the chromosomal localisation of 3q 28-29. Taking into account the common abnormalities of chromosome 3 described in UM cells, ranging from complex rearrangements/deletions at chromosome 3q, such as in OCM-1 cells (Luyten *et al*, 1996; Nareyeck *et al*, 2006), to complete loss of a chromosome (monosomy 3), we screened the UM cell lines for the level/presence of p63 protein. Our results showed very low or lack of p63, both at the transcript and protein level, suggesting the absence of functionally active p63 protein in these cells, most likely affected by the chromosomal abnormalities.

The role of p63 as a key player in the development and maintenance of stratified epithelia has been extensively studied and is widely accepted. However, the apoptotic and tumour-suppressive role of p63, mainly driven by its isoforms containing transactivation domain (TAp63), has remained more enigmatic. Although p63 and p73 can induce apoptosis in response to DNA damage, the notion that p53-induced apoptosis requires the participation of p63 and p73 family members remains controversial. By using the E1A programmed murine embryo fibroblasts, Flores *et al* (2002) showed that the combined loss of p63 and p73 resulted in the failure of cells exhibiting functional p53 to undergo apoptosis in response to DNA damage (Flores *et al*, 2002). In another study, it was shown that the TAp63 isoform is essential in the process of DNA damage-induced oocyte death, a process that previously had been thought to be mediated by p53 (Suh *et al*, 2006). Furthermore, the induced oocyte death involved the phosphorylation of p63 and its binding to p53 cognate DNA sites. However, there are studies indicating that at least in thymocytes, p53-dependent apoptosis occurs independently of p63 and p73 (Senoo *et al*, 2004) and that these p53 homologues are not required for p53-mediated lymphoma suppressor activity (Perez-Losada *et al*, 2005). In the present study, the OCM-1 cells displayed high resistance towards apoptosis, despite the presence of functional p53. The treatment with even high doses of TRAIL as well as UV irradiation failed to induce apoptosis in OCM-1. However, these apoptosis-resistant cells readily committed to apoptosis when p63 levels were increased following overexpression of p63. The elevated levels of p63 induced transcription of PERP in OCM-1 cells, which was not possible by the expression of p53 alone. These findings are strongly supported by previous studies in which no association was observed between p53 and PERP promoter in p63^−/−^ E1A cells in response to DNA damage. p63 was found to bind to both the 5′ and 3′ binding sites on the PERP promoter (being more strongly associated with 5′ site). By contrast p53 was detected only at the 3′ site. Significantly, p63 was also shown to bind to the PERP promoter even in the absence of p53 (Flores *et al*, 2002).

p63 expression levels were transient in our study with a gradual increase from 16 to 24 h, followed by a subsequent reduction. PERP transcription was strongly associated with the expression levels of p63 and decreased after 24 h, indicating that p63 may function through an auto-regulatory feedback loop, in which transcriptional activity of p63 could be directly linked with its degradation or inhibition. Moreover, in our study, p53 and p63 displayed a similar expression profile with a transient increase followed by a decrease in the protein levels. Given the similarities between the two proteins, they might be regulated, at least in part, through common pathways. p53 levels are controlled in large part by MDM2, which acts either by inhibiting the transactivation function (Momand *et al*, 1992; Oliner *et al*, 1993) or by promoting ubiquitin-mediated degradation (Haupt *et al*, 1997). MDM2 can also bind to the N-terminus of p63 and has been variously reported to inhibit the p63 transactivation function (Kadakia *et al*, 2001) or to have no effect (Little and Jochemsen, 2001). Another mode of inhibition has also been demonstrated for p63 through an intramolecular mechanism. p63 *α* isoforms exhibit a 27 kDa C-terminal region that is both necessary and sufficient for transcriptional inhibition and acts by binding to the region in the N-terminal of the TA domain of p63 that is homologues to MDM2 binding site in p53, hence masking residues that are important for transactivation (Serber *et al*, 2002).

In summary, this study highlights for the first time the p63 requirement for initiation of apoptosis in UM and provides the experimental evidence of its essential function alongside p53 in the PERP-mediated apoptosis. Although our findings do not diminish the significance of p53 in this process, they do underscore the importance of p63 in the p53-dependent apoptotic and tumour-suppressive function of PERP. Furthermore, the findings provide for the first time a functional link between a molecular determinant (p63) highly likely to be affected by the well-described alterations of chromosome 3 in UM and the pathogenesis of this tumour. The findings have also broad-ranging implications for other cancers in which similar overall dysfunction of p63 may underlie the regulation of apoptosis effectors such as PERP and the impaired ability of tumour cells to engage in apoptosis. Such insights are expected to prove advantageous in designing therapeutic approaches for increasing susceptibility to apoptosis required to overcome the tumour resistance to chemotherapy and radiotherapy.

## Figures and Tables

**Table 1:** Gene-specific primer sets for real-time qPCR

**Gene**	**Primer sequence**	**Annealing temperature (°C)**
*PERP*	5′-CCAGATGCTTGTCTTCCTGAGAG-3′ 5′-AGTGACAGCAGGGTTGGCATGA-3′	65
*p63*	5′-CAGGAAGACAGAGTGTGCTGGT-3′ 5′-AATTGGACGGCGGTTCATCCCT-3′	65
*GAPDH*	5′-AACAGCCTCAAGATCATCAG-3′ 5′-TGAGTCCTTCCACGATACC-3′	60

Abbreviation: qPCR=qunatitative PCR.
